# Extracellular Vesicles in the Development of Cancer Therapeutics

**DOI:** 10.3390/ijms21176097

**Published:** 2020-08-24

**Authors:** Haoyao Sun, Stephanie Burrola, Jinchang Wu, Wei-Qun Ding

**Affiliations:** 1 Department of Pathology, University of Oklahoma Health Science Center, Oklahoma City, OK 73104, USA; haoyao-sun@ouhsc.edu (H.S.); Stephanie-Burrola@ouhsc.edu (S.B.); 2 Department of Radiation Oncology, The Affiliated Suzhou Hospital of Nanjing Medical University, Suzhou 215001, China; 3 Section of Oncology, The Second Affiliated Hospital of Xuzhou Medical University, Xuzhou 221006, China

**Keywords:** extracellular vesicle, microvesicle, exosome, cancer therapeutic, drug carrier

## Abstract

Extracellular vesicles (EVs) are small lipid bilayer-delimited nanoparticles released from all types of cells examined thus far. Several groups of EVs, including exosomes, microvesicles, and apoptotic bodies, have been identified according to their size and biogenesis. With extensive investigations on EVs over the last decade, it is now recognized that EVs play a pleiotropic role in various physiological processes as well as pathological conditions through mediating intercellular communication. Most notably, EVs have been shown to be involved in cancer initiation and progression and EV signaling in cancer are viewed as potential therapeutic targets. Furthermore, as membrane nanoparticles, EVs are natural products with some of them, such as tumor exosomes, possessing tumor homing propensity, thus leading to strategies utilizing EVs as drug carriers to effectively deliver cancer therapeutics. In this review, we summarize recent reports on exploring EVs signaling as potential therapeutic targets in cancer as well as on developing EVs as therapeutic delivery carriers for cancer therapy. Findings from preclinical studies are primarily discussed, with early phase clinical trials reviewed. We hope to provide readers updated information on the development of EVs as cancer therapeutic targets or therapeutic carriers.

## 1. Introduction

Extracellular vesicles (EVs) are a generic term referring to several groups of small lipid bilayer-delimited particles generated through various cellular processes and released from all types of cells investigated thus far. These membrane vesicles, including microvesicles (also known as microparticles or ectosomes), exosomes, and apoptotic bodies, all lack a functional nucleus and are unable to replicate themselves. They are constantly released from cells and are involved in a variety of physiological as well as pathological processes. The initial discovery of EVs can be tracked back to 1946 when ultracentrifugation pellets were found to be associated with the activation of platelets and procoagulant properties in human plasma [1]. In the 1980s, EVs released by reticulocytes were captured by electronic microscopy and were considered “waste disposals” to remove waste materials during red blood cell maturation [2,3]. However, EV-mediated transfer of genetic and cellular materials between different cell types was recognized in the late 2000s by several research groups [4,5,6,7,8], thus establishing EVs as messengers for intercellular communication with biological consequences.

Among all the EVs described, exosomes are defined by their small sizes (40–120 nm) and endocytic origin and are most extensively characterized over the years. In the context of cancer, it has been demonstrated that exosomes play a pivotal role in the tumor microenvironment by mediating intercellular communication among cancer cells and stromal cells, thereby promoting tumor proliferation, metastasis, and chemo-resistance [9]. The contribution of exosomal signaling to tumor progression has led to the development of therapeutic strategies targeting various steps of the exosomal signaling processes (see Section 2). On the other hand, since exosomes are endogenously produced and can be transferred among various types of cells, the potential of using these small vesicles as vehicles for drug delivery has been actively explored (see Section 3). In this review, we will focus on recent work in the development of cancer therapeutics targeting EV-mediated cellular processes or utilizing EVs as vehicles for drug delivery. Furthermore, we will discuss the clinical trials that are ongoing or completed using naturally produced EVs as cancer therapeutic vehicles. A simplified view of general aspects of EVs is provided at the first section of this review.

## 2. EV Cargos and Functions

### 2.1. EV Nomenclature

EVs were initially called platelet dust, as they were vesicles derived from platelets. In the 1970s, the term “extracellular vesicles” was used to describe calcifying globules in epiphyseal cartilage that were observed by histochemical staining [10]. Since then, the nomenclature of EVs has significantly evolved and EVs are now named primarily according to their sizes and biogenesis processes or the way of release [11]. It is well accepted that there are three main subgroups of EVs that have been identified thus far: (a) exosomes, (b) microvesicles (MVs, also named microparticles/ectosomes), and (c) apoptotic bodies [12]. The most researched EVs are exosomes, which were firstly termed in the 1980s as a group of vesicles ranging from 40 to 120 nm in diameter, formed by the invagination of the multi-vesicular bodies (MVBs) during the late endosome formation [2,3,13]. Differing from exosomes, MVs are larger membrane vesicles (up to 1000 nm in diameter) which are produced by direct budding from cellular membranes, whereas apoptotic bodies are even larger vesicles with 800–5000 nm in diameter and formed during programmed cell death [14,15]. Recently, a smaller group of non-membranous nanoparticles termed “exomeres” (~35 nm) was also reported, which is likely to be generated through a unique cellular process [16]. The overlap in sizes of different EV groups and the difficulty in separating individual EV groups by current isolation techniques have hindered our understanding of their biogenesis, molecular compositions, biodistributions, and functions. For this reason, the International Society for Extracellular Vesicles (ISEV) provided guidelines on the terminology and minimum requirements for defining EV populations in experimental research in 2014, which was updated in 2018 (MISEV2018) [17]. Most notably, instead of using the terms exosomes or MVs, the guidelines urge authors to name EV subtypes based on their physical characteristics, such as size or density, with ranges being defined, biochemical compositions, and the experimental conditions or cell of origin. In accordance with this recommendation, exosomes are considered small EVs (sEVs), which is the term we used interchangeably with exosomes, wherever appropriate, throughout this review.

### 2.2. EV Surface Markers and Cargos

EVs carry various biomolecules including proteins, RNA, DNA, and lipids. Each group of biomolecules in EVs is often heterogeneous, primarily relating to different EV types, experimental conditions, and their cellular origins [11]. The most characterized EV components are EV proteins and RNAs, especially small RNAs [18]. EV surface protein markers have been critically examined in order to establish specific markers for validating isolated EVs. The MISEV2018 guidelines provide several groups of protein markers in evaluating isolated EVs as well as minimal requirements in experimental data presentation when it comes to EV isolation and characterization [17].

It has come to a consensus that sEVs stably express specific transmembrane proteins such as tetraspanins (most notably CD63, CD9, CD81), Major Histocompatibility Complex (MHC) class I proteins (such as HLA-A/B/C), transferrin receptor, LAMP1/2, and others. These membrane proteins, especially tetraspanins, are frequently applied to validate isolated sEVs. In addition, cytosolic proteins can also be specific markers for sEVs, including Alix, TSG-101, flotillins-1/2, annexins, and heat shock proteins, among others. Cell- or tissue-specific EV markers have also been reported, such as TSPAN8 and EPCAM (epithelial cell), CD37 and CD53 (leukocytes), PECAM1 (endothelial cells), and ERBB2 (breast cancer). Given the heterogeneity of EVs, it is recommended that at least one membrane protein marker, one cytosolic protein marker, and one non-EV protein marker have to be used to validate the isolated sEVs from large EVs [16,17]. It has been recognized that proteins from the nucleus, mitochondria, endoplasmic reticulum, and the Golgi complex are mostly absent in sEVs, which can serve as negative control markers for these vesicles [19]. Enormous efforts have been placed on profiling proteomes of sEVs and the comprehensive databases of sEV proteins can be found at: Vesiclepedia [15], EVpedia [20], and ExoCarta [21].

sEVs contain various RNA species. However, most studies demonstrated that small non-coding RNAs, such as microRNAs, are the major RNA species contained in sEVs, although the presence of mRNA, rRNA, and tRNA in sEVs was also reported [22,23]. Typically, sEVs may contain hundreds of microRNA species in various quantities that play important roles in intercellular communication [7,23,24,25]. Both coding and non-coding RNAs seem to be functional through transferring from host cells to the recipient cells [26,27,28]. Specific RNA profiles of sEVs derived from different biofluids or tissues are categorized by several databases, including: Exobase [29], exRNA Atlas [30], and miRandola [31].

DNA in sEVs has also been described, with DNA fragments originating either from the nucleus or from the mitochondria. It seems that all genome DNA are represented randomly in sEVs, which eliminates the possibility of selective DNA packaging [32,33,34]. While cancer cell-derived sEVs may contain more genomic DNA than that from non-cancer cells [34], whether and how sEV DNA contributes to intercellular communications in the tumor microenvironment, thereby affecting tumor progression, remains to be determined.

### 2.3. EV Functions

It has long been known that cell-to-cell communication is a strategy utilized to facilitate physiological and pathological processes in various organisms. However, the EV-mediated intercellular communication was only recognized in recent years [7,23]. The double-layer lipid membrane of EVs protects inside contents, allowing transfer of EV materials to surrounding cells or to distal organs via the circulatory system. Most notably, sEVs have been considered potent vehicles to mediate intercellular communication [11]. By transferring signaling molecules among different cell types, sEVs have been shown to play pleiotropic roles in regulating cellular and physiological processes. This includes participating in hemostasis by enhancing coagulation, regulating both innate and acquired immune responses, involvement in pregnancy and embryonic development, as well as other physiological events [35,36,37,38,39,40,41,42,43,44].

In addition to mediating intercellular communication, EVs may function as waste disposals to remove unwanted cellular materials. In fact, sEVs were first observed to facilitate reticulocyte maturation via cargo disposing [2,3]. In supporting this function of sEVs, several recent studies revealed the cross-regulation of the EV pathway and lysosomal degradation pathway [45]. Two established lysosome inhibitors, chloroquine and bafilomycin A1, were shown to enhance sEV release [46,47,48], suggesting that sEVs may act as an alternate pathway for cell component degradation and clearance. The involvement of sEVs in cellular homeostasis is further supported by the findings showing that ubiquitin and ubiquitinated proteins are present in sEVs [49], along with selective lipids and other soluble cellular components [50,51].

The role of sEVs in pathological processes has been evident, especially in cancer. Cancer progression is a dysregulated and uncontrolled pathological process [52]. It is well described that cancer-derived sEVs promote tumor development [53,54,55] by acting at different stages of cancer progression [56] through various mechanisms. Evidence is provided to indicate that cancer sEVs are involved in enhancing tumorigenesis of epithelial cells [53,57], sustaining tumor angiogenesis [58,59], promoting tumor growth [60,61], facilitating cancer cell invasion and metastasis [54,55,62,63], and contributing to chemo-resistance [64,65] and immunosuppression [66,67]. These important findings of the tumor-promoting effects of cancer sEVs lead to new cancer therapeutic opportunities that aim at targeting cancer exosomal signaling processes, as discussed below.

## 3. EVs as Potential Therapeutic Targets in Cancer

Given the growing evidence of sEVs’ involvement in cancer progression, several strategies have been tested or envisioned to target various steps of the sEVs signaling in order to block their tumor promoting effect. These include targeting cancer sEV biogenesis and release, blocking sEV uptake by recipient cells, eliminating circulating cancer sEVs, and removing specific components from sEVs that contribute to cancer pathogenesis [68,69,70].

### 3.1. Suppressing sEV Biogenesis and Release

At the cellular level, sEVs are derived from the endosomal pathway. The invagination of endosomal membranes generates intraluminal vesicles inside of the endosome, forming MVBs. These vesicles are released by cells upon fusion of the endosome with the cellular plasma membrane and the released vesicles are termed exosomes or sEVs [71,72]. The process of forming sEVs and releasing them from cells requires a coordinated effort by various cytoplasmic proteins. This includes endosomal sorting complexes required for transport (ESCRT) and tetraspanins necessary for intraluminal vesicle formation, sphingomyelinase to generate ceramides vital for intraluminal vesicles’ formation and sorting, and Rab27a and Rab27b critical for cellular endosomal trafficking [55,71,73]. In an early effort to suppress sEVs’ biogenesis, GW4869, a sphingomyelinase inhibitor, was used, which reduced ceramide generation and inhibited sEV formation [74]. Furthermore, attenuation of neutral sphingomyelinase 2 (nSMase2) in breast cancer cells by a knockdown approach reduced sEV formation and attenuated sEV-associated miR-210 transfer, leading to the suppression of cancer cell metastasis in vitro and in a xenograft mouse model [75]. However, the role of nSMase2 in sEV formation and secretion from other cultured cancer cell lines remains unclear [76,77], compromising this approach of targeting sEV biogenesis. Other potential strategies in targeting sEV biogenesis that have been tested or envisioned include the use of Amiloride, an anti-hypotension drug, which reduced sEV yields by blocking membrane-associated heat shock protein 72 (HSP72) in a STAT3-dependent manner in myeloid-derived suppressor cells [78]; inhibiting the syndecan-syntenin-Alix signaling process, since the syndecan heparan sulphate proteoglycans and their cytoplasmic adaptor syntenin, along with Alix and ESCRT, control the formation of sEVs [79]; and targeting cellular molecules, such as Rab27a/b [70,73,80], Rab11, Rab35 [81,82], TSG101, and TSAP6 [70], which are either related to sEV formation or trafficking and secretion from cancer cells. Using a high-throughput screening approach, a recent study identifies that manumycin-A (MA), a natural microbial metabolite, inhibits sEV biogenesis and secretion via the Ras/Raf/ERK1/2 signaling in castration-resistant prostate cancer cells but not in normal prostate epithelial cells [83], indicating a new compound that may serve as a cancer therapeutic via inhibiting sEV biogenesis and secretion. In another high-throughput screening study, miR-26a was identified as being involved in sEV secretion from prostate cancer cells [84], suggesting a new molecular target for suppressing cancer sEV secretion.

### 3.2. Preventing EV Uptake

**Figure 1:**
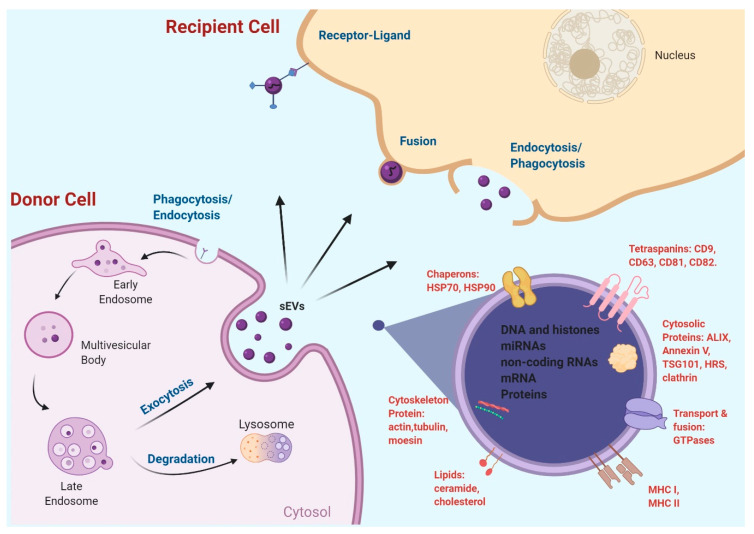
sEV biogenesis, release, uptake, and contents. Created with BioRender.com.

Several sEV uptake mechanisms have been recently proposed (Figure 1), including sEV membrane direct fusion with plasma membrane, thereby releasing sEV contents to recipient cells [85,86], and receptor-mediated endocytosis [87], clathrin- and caveolin-mediated endocytosis [88,89], phagocytosis [90], and macropinocytosis [88,91]. Detailed regulation of each of the pathways and their proportional contributions to sEV uptake remains to be further elucidated. It seems reasonable to assume that the uptake to a large extent depends on sEV surface protein compositions and the type of cells in which the sEVs are internalized. Furthermore, irrespective of the uptake pathways, internalized materials will be processed via the endosomal/lysosomal pathway [92]. While limiting cancer sEV uptake by recipient cells is a potential strategy to block cancer sEV signaling and attenuate cancer sEVs’ tumor-promoting effect, few studies have been published to support this strategy. Nevertheless, evidence has been provided to indicate that it is feasible to modulate the sEV uptake process in order to attenuate the sEV-induced effect in the recipient cells. Some examples include the following. Autophagy inhibitors such as chloroquine, bafilomycin A, and monensin, were shown to significantly inhibit sEV internalization into microglia, likely through altering vacuolar acidification [91]. Two potent PI3K inhibitors, Wortmannin and LY294002, concentration-dependently inhibited sEV uptake by macrophages, indicating that PI3K is essential for sEV phagocytosis [90]. Disruption of the actin cytoskeleton using Cytochalasin D or Lantrunculin A inhibited sEV uptake by Human Umbilical Vein Cells (HUVECs), confirming that an intact cytoskeleton facilitates sEV internalization [93]. Chlorpromazine, which blocks clathrin-mediated endocytosis, inhibited sEV uptake by ovarian cancer cells [94] and endothelial cells [95], and heparin dose-dependently inhibited sEV uptake by glioblastoma (GBM) cells [96] and bone marrow stromal cells [97]. These findings reinforce the notion that targeting the uptake of cancer sEVs is a promising strategy in the development of new cancer therapeutics, and future efforts should focus on small molecules capable of inhibiting cancer sEV uptake and suppressing tumor progression.

### 3.3. Eliminating Circulating Cancer sEVs

The transfer of cancer sEVs through the circulatory system to distal organs has been reported to promote tumor metastasis via various mechanisms, most notably by forming pre-metastatic niches in the distal organs [55,62,98]. Considering that most cancer deaths are due to metastatic disease, eliminating circulating cancer sEVs is presumably a great strategy to prevent cancer metastasis, thereby reducing cancer mortality. The idea of “cleaning” the blood to prevent cancer metastasis has been tested many years ago. In the late 1980s, using a continuous whole blood UltraPheresis procedure, plasma fractions with molecular weight less than 150 kDa were removed from patients with metastatic cancer, which reduced tumor size and improved patient immune response and Karnofsky Performance Status [99]. While this technique did not consider removing blood sEVs at the time, it inspired others to develop new devices to remove cancer sEVs from patient plasma [64]. For instance, Hemopurifier^®^, an affinity-based purifier developed by Aethlon Medical Inc. (San Diego, CA, USA), has been shown to selectively capture viral particles (which have similar size as sEVs) in the plasmas of individuals infected with Hepatitis C and Human Immunodeficiency Virus (HIV) [100,101], and this device is being modified and tested for removal of Her2-positive breast cancer exosomes from patient plasma ([64], https://grantome.com/grant/NIH/R43-CA232977-01). Moreover, a phase I clinical trial using Hemopurifier^®^ in conjunction with pembrolizumab (Keytruda) in patients with advanced head and neck cancer has been recently approved by the Food and Drug Administration (FDA) (NCT04453046).

In line with the strategy of eliminating circulating cancer sEVs, a recent report demonstrated, in a xenograft nude mouse model, that treatment of the mice with human anti-CD9 and anti-CD63 antibodies (intravenous injection) disrupts cancer sEVs in the circulation and suppresses the pulmonary metastasis of implanted human breast cancer cells, yet, has no effect on primary tumor growth of the implants or metastatic ability of the cells in vitro [102]. These findings support the strategy to suppress cancer metastasis via inhibiting the pro-metastatic functions of cancer-derived sEVs using antibodies against their surface proteins. In addition, an innovative design of aptamer-functionalized nanoparticles was shown to eliminate blood oncogenic sEVs into the small intestine, and attenuate oncogenic sEV-induced lung metastasis in mice [103]. This technology utilized positively charged mesoporous silica nanoparticles equipped with Epidermal Growth Factor Receptor (EGFR)-targeting aptamers specifically recognizing and binding the negatively charged oncogenic sEVs and towing them from blood to bile duct for elimination. This interesting study proves that it is feasible to remove oncogenic sEVs selectively from the blood stream, thereby reducing tumor metastatic potential. Further investigations are warranted along this line of research.

### 3.4. Targeting Specific sEV Cargo Components

Specific sEV components that mediate sEVs’ tumor-promoting activity are obvious potential cancer therapeutic targets. Some of the targets have been recently explored in order to develop new cancer therapeutics. As discussed above, antibodies against human CD9 and CD63, two well-established sEV surface markers [17], were shown to disrupt oncogenic sEVs and inhibit tumor metastasis in a breast cancer xenograft nude mouse model [102]. However, this experiment strategy of targeting human CD9 and CD63 is only applicable in a xenograft nude mouse model for selectively eliminating human cancer sEVs from the blood, since CD9 and CD63 are expressed in sEVs released from both noncancerous and cancerous cells in humans. Targeting of cancer-specific sEV components will be preferred to achieve a cancer-specific effect. In this context, a recent report demonstrated that cytoskeleton-associated protein 4 (CKAP4), a novel Dickkopf1 (DKK1) receptor, was selectively contained in sEVs from pancreatic ductal adenocarcinoma (PDAC) cells, not in sEVs from normal cells. Various anti-CKAP4 antibodies were then utilized to block the interaction of DKK1 with sEV-associated CKAP4, resulting in an inhibition of the proliferation and migration of PDAC cells and a prolonged survival of PDAC xenograft nude mice [104], supporting further development of this targeting strategy.

In another report, miR-365 in macrophage-derived sEVs was found to significantly decrease the sensitivity of PDAC cells to gemcitabine, and a miR-365 antagonist was able to reverse the gemcitabine resistance of PDAC cells in vitro and in vivo [105], thus suggesting that targeting miR-365 in macrophage-derived sEVs renders PDAC cells more sensitive to gemcitabine. Similarly, miR-155 was found in PDAC cell-derived sEVs that mediates transfer of the gemcitabine resistance traits from resistant PDAC cells to sensitive PDAC cells, conferring gemcitabine resistance of PDAC cells. Targeting miR-155 or the exosome secretion of PDAC cells effectively attenuated the gemcitabine resistance in PDAC cell lines and in xenograft nude mice [106]. Other cancer sEV-associated microRNAs, such as miR-21 and miR-1246, have also been found to be selectively enriched in cancer sEVs and considered as therapeutic targets [107,108]. Since cancer sEVs selectively encapsulate certain microRNA species [25,109,110,111], targeting cancer sEV-associated microRNAs will continue to be an attractive strategy for the development of new cancer therapeutics.

Immune checkpoint protein inhibitors, such as PD1/PD-L1 inhibitors, are novel cancer therapeutic targets which have revolutionized cancer therapy with great efficacy, even for those cancer patients whom standard therapy has failed [112]. However, only 10%–30% of patients responded to checkpoint inhibitor therapy [113]. The immune escape is partially due to the fact that tumor-derived sEVs contain PD-L1, a PD1 ligand, which binds to PD1 on the surface of T cells and suppresses T cell activation [66,67]. The sEV PD-L1 level was thus suggested to be a prognostic marker for anti-PD1 therapy response [114], and blocking sEV PD-L1 has been proposed to overcome the resistance to anti-PD-1/PD-L1 antibody therapy [115]. Indeed, anti-PD-L1 antibodies were shown to block sEV PD-L1, induce systemic anti-tumor immunity, and suppress tumor growth in a syngeneic colorectal cancer model [67].

New oncogenic components in cancer sEVs are continuously being identified which may contribute to tumor progression or chemo-resistance [116,117]. Efforts on targeting these sEV-associated oncogenic molecules for cancer therapeutic development are expected to expand.

## 4. EVs as Drug Carriers in Cancer Treatment

Compared to artificial drug vehicles, such as liposomes, EVs are favored drug carriers [118] because of their autologous nature that would prevent undesired immunogenicity and toxicity [119,120]. sEVs also possess high capacity of homing toward tumor cells when compared to liposomes [62,121], implying that sEVs are more efficient in delivering drugs for cancer therapy. Furthermore, studies have shown that sEVs are stable membrane vesicles under different pH values, temperatures, or freeze–thaw cycles [122], and these properties can be further enhanced by surface modification [123], supporting their potential compliance with good manufacturing practices (GMPs) in future clinical use. In addition, as nano-sized particles, sEVs were shown to be able to cross the blood–brain barrier and the tumor vasculature via enhanced permeability and retention (EPR), thereby potentially increasing accumulation of nanoparticles in brain tumors [124,125,126].

Diverse techniques have been practiced to encapsulate cancer therapeutics by sEVs in order to develop more efficient tumor-targeting vehicles. Here, we review the sEV loading strategies reported in recent literature.

### 4.1. EV Sources and Loading Efficiency

Based on the heterogeneity of sEVs derived from various biological sources [18], it is safe to assume that the source of the sEVs may relate to their drug loading efficiency and their therapeutic efficacy. Indeed, experimental evidence has been provided to show that drug loading efficiency of sEVs derived from pancreatic stellate cells (PSCs), pancreatic cancer cells (PCCs), and macrophages significantly differ when doxorubicin was simply incubated with the sEVs, with those from PCCs being most efficient. However, the doxorubicin-loaded macrophage sEVs are most effective in killing cancer cells, indicating that higher loading capacity does not equal to high anticancer activity of the drug-loaded sEVs [127]. This implies that both the biological source of the sEVs and the drug loading efficiency need to be evaluated when sEVs are applied as drug carriers for cancer therapy. In line with this concept, sEVs derived from mesenchymal stem cells (MSCs) are considered good carriers for drug delivery because they possess low immunogenicity [9,128] and are well tolerated in mice [129] and humans [130]. Both a miR-9 inhibitor and the chemo drug paclitaxel have been successfully incorporated into sEVs derived from MSCs which inhibited tumor cell growth [131,132]. However, allogeneic MSCs may also be able to transfer immunogenic proteins, such as MHC molecules, via secreted EVs, which might cause immunological responses [133]. Furthermore, the immunogenicity of MSCs-derived EVs varies, depending on experimental conditions by which the EVs are produced [134]. Future efforts are required to closely monitor immunologic responses post administration of MSCs-derived EVs and develop uniform procedures in preparing MSCs-derived EVs. In addition to MSCs, sEVs from immature dendritic cells or self-derived dendritic cells were also considered, possessing low immunogenicity and used to encapsulate siRNA or doxorubicin for therapeutic applications [135,136]. Interestingly, cancer cell-derived sEVs were shown to have unique targeting abilities homing to tumorous microenvironments [137]. sEVs from HeLa and patient ascites were shown to deliver heterologous siRNAs to HeLa cells and cause cell death [138]. Autologous sEVs were found to be safe and effective in delivering gemcitabine for pancreatic cancer therapy in experimental model systems [139]. These results show that cancer cell-derived sEVs are promising carriers for effective delivery of chemotherapeutic drugs or nucleotides. Given the tumor-promoting activity of cancer-derived sEVs [53,54,55], the safety and long-term effect of these membrane vesicles as drug-delivery carriers needs to be carefully evaluated.

### 4.2. Loading Therapeutics into sEVs via Donor Cells

Efficient loading of cancer therapeutics into a given sEV population can be critical when it comes to drug efficacy. In this context, one loading strategy that has been described in packaging cancer therapeutics into sEVs is to load cancer therapeutics into sEVs via donor cells, which is in contrast to directly loading therapeutics into isolated sEVs. In this case, microRNAs have been most often loaded into sEVs via the donor cells. For example, adipose tissue-derived MSCs were transfected with a miR-122 expression plasmid to overexpress this microRNA and the sEVs derived from these cells were highly enriched in miR-122. An intra-tumor injection of miR-122-enriched sEVs significantly increased the efficacy of Sorafenib on inhibiting hepatocellular carcinoma in a xenograft nude mouse model [140]. Functional delivery of miR-21 derived from glioma cells to the surrounding microglia led to downregulation of specific miR-21 mRNA target genes [141]; likewise, sEVs from primary glioma cells, stably expressing miR-302-367, were shown to enrich in miR-302-367 by internalizing neighboring glioblastoma cells, and altering tumor development in vivo [142], and overexpression of miR-146b in marrow stromal cells generated sEVs with high miR-146b content, which significantly reduced glioma xenograft growth in rats [143]. More studies have been reported in testing the strategy of loading microRNA inhibitors or mimics into sEVs via the donor cells for therapeutic applications, as was recently reviewed [144].

An interesting study demonstrated that the chemotherapeutic paclitaxel (PTX) could be added directly to the culture of MSCs to generate sEVs that are highly associated with PTX and significantly suppress cancer cell proliferation [132]. However, this strategy of loading chemotherapeutics into sEVs has been less explored, likely because of the loading efficiency, considering the potential metalizing of PTX in treated cells. Instead, direct loading of chemotherapeutics and microRNA/siRNAs into the isolated sEVs has been widely adapted for testing sEVs as drug carriers for therapeutic delivery.

### 4.3. Loading Therapeutics into Isolated sEVs

The lipid-bilayer membrane structure of sEVs favors encapsulating hydrophobic compounds and molecules, which may directly integrate into the sEVs without disturbing their membrane barrier. In contrast, hydrophilic compounds and molecules require permeabilization of the bilayer membrane in order to be incorporated into the sEVs [145,146]. Various approaches have been proposed to load hydrophobic and hydrophilic drugs or biological molecules into sEVs. The most common approaches include opening up the pores in lipid membranes by physic forces, such as electroporation, sonication, freeze and thaw cycles, and extrusion, and by chemical means, such as using transfection reagents. The pros and cons of these methods for membrane permeabilization and cargo loading has been reviewed elsewhere [147]. Therefore, we will only briefly discuss these loading approaches in the following.

Direct incubation of therapeutics with sEVs at given temperatures and durations is a simple strategy for loading drugs into sEVs. The loading efficiency mainly relies on the concentration of the drugs or molecules and their hydrophobicity. A proper loading can usually be achieved for hydrophobic compounds without disturbing the integrity of the sEV membrane [132]. Nevertheless, the loading efficiency is often lower compared to other loading approaches.

Electroporation has been a method widely used to introduce DNA or RNA into mammalian cells [148,149], and is often applied for drug or nucleotide loading into sEVs [150,151]. The desired sEVs will be co-incubated with the therapeutics and exposed to certain volts of electric fields to open up the pores of the sEV membrane to allow the therapeutics to enter into the permeabilized sEVs. This method has been preferentially applied when incorporating nucleic acids like siRNA, mRNA, DNA, and microRNA, into sEVs [152]. Its loading efficiency is usually higher than incubation [139]. However, the main drawback of this method is the risk of damaging the EV membranes that may cause aggregation of sEVs and precipitation of nucleic acids.

Sonication uses ultrasound energy transmitted through a sonicating probe that reduces the rigidity of sEV membranes, thus allowing more therapeutic molecules to be incorporated into sEVs. For example, PTX was loaded into sEVs more efficiently by sonication than electroporation and incubation [139]. However, the sonicating probe produces consistent heat during the sonication and the operation has to be done on ice, with intervals between strokes [153]. There is no doubt that sonication may compromise the membrane integrity of sEVs, with the therapeutics occasionally being attached to the outer membrane of the sEV, which affects the drug distribution in vivo [139].

The freeze and thaw approach takes advantage of the formation of ice crystals that temporarily disrupt the sEV membrane, allowing therapeutic compounds to enter into the sEVs prior to membrane reconstitution [154]. This method shows lower cargo loading compared to sonication- and extrusion-based methods [155]. One to three cycles of freeze and thaw were usually performed during drug incorporation, which may accelerate the degradation and aggregation of the sEVs [122,156].

Extrusion utilizes a lipid syringe extruder with pore sizes between 100 and 400 nm, which break the sEV membrane physically and then mix with therapeutics. This method possesses high loading efficiency when compared to freeze and thaw, sonication, and saponin treatment [155,157]. One can imagine that the extrusion approach may cause damage of the sEV membranes as it does by sonication and electroporation.

Saponin treatment and the use of common transfection reagents, such as cationic lipids, have also been applied to load exogenous materials into sEVs. It was demonstrated to be an effective approach for sEV encapsulation of therapeutic drugs when compared to electroporation [155]. While we would expect more studies using the transfection approach for sEV loading, especially for loading of nucleic acids, the chemical transfection reagent itself will need to be removed prior to delivering the sEVs to target cells [157].

Through the above-mentioned approaches, multiple therapeutic agents in the forms of DNA, microRNA, siRNA, porphyrins, proteins (catalase, stress-induced heat shock proteins), and chemotherapeutics (curcumin, paclitaxel, docetaxel, gemcitabine) have been successfully loaded into sEVs and tested for their therapeutic value [158,159,160,161]. Nonetheless, it remains to be determined which approaches are most appropriate for loading specific agents into desired sEVs.

## 5. Clinical Trials Testing sEVs as Cancer Therapeutic Carriers

The potential of sEVs to serve as cancer therapeutic carriers and the promising results from preclinical studies have led to clinical trials aimed to develop sEV-based cancer therapy. We searched ClinicalTrials.gov and Pubmed.gov on 7 July 2020 and found 12 clinical trials testing sEVs as potential cancer therapeutics or therapeutic carriers, with 8 of them being registered in ClinicalTrials.gov (Table 1). Some of the clinical trials have reported their end results and others are still ongoing [162]. These clinical trials can be categorized according to their biological source of sEVs that are used as therapeutic carriers, as discussed below. Note that these clinical trials are mostly in early stages, and the definitive therapeutic value of sEVs for cancer therapy has yet to be determined.

### 5.1. Clinical Trials Using Dendritic Cell-Derived sEVs (DEX)

In 2005, two phase I clinical trials were reported using autologous DEX as immune stimulants, one for patients with metastatic melanoma, and another for patients with non-small cell lung cancer (NSCLC) [169,170]. Similar procedures were used in isolating sEVs from patients and loading MAGE-3 antigens to the sEVs for these trials. In the metastatic melanoma trial, 15 patients were included and received a 4-week outpatient vaccination course with antigen-loaded DEX given intradermally (1/10th) and subcutaneously (9/10th) per week for 4 weeks. There was no major toxicity being observed and some patients showed partial response and tumor repression. This is the first study to show the feasibility and safety of DEX-based vaccination in melanoma patients. In the NSCLC trial, 13 patients were enrolled, with 9 completing the therapy. The antigen-loaded DEX was given, intradermally (1/10th) and subcutaneously (9/10th), 4 times at weekly intervals. Similar to the melanoma trial, no major toxicity was observed during a 24-month follow up, and immune activation and stability of disease was observed in some patients with advanced NSCLC. The success of this phase I trial led to a phase II clinical trial for NSCLC in France (NCT01159288). In the phase II trial, DEX was upgraded from the first-generation interferon gamma-free DEX (IFN-γ-free DEX) to a second generation (IFN-γ-DEX) in order to enhance DEX-induced T cell responses. Twenty-four patients were recruited, and the results confirmed that DEX boosts antitumor immunity in patients with advanced NSCLC with outstanding safety [168]. Together, these clinical trials indicate a potential safe immunotherapy using DEX in metastatic melanoma and NSCLC, and an enhanced efficacy of DEX when administered in combination with IFN-γ.

### 5.2. Clinical Trials Using Ascites-Derived sEVs (AEX)

In 2008, a phase I study using autologous AEX combined with granulocyte-macrophage colony-stimulating factor (GM-CSF) for colorectal cancer was completed [167]. Forty patients with advanced colorectal cancer were included in the study and randomly assigned to AEX alone or AEX plus GM-CSF groups. Patients received 4 subcutaneous immunizations at weekly intervals. Results showed that both groups of patients tolerated the treatment well and AEX plus GM-CSF rather than AEX alone induces beneficial antitumor cytotoxic T lymphocyte (CTL) response. These findings suggest that the immunotherapy of colorectal cancer with AEX in combination with GM-CSF is feasible and safe, and may be applied for immunotherapy of colorectal cancer.

### 5.3. Clinical Trials Using Tumor Cell-Derived EVs

A preclinical study has confirmed the feasibility of using apoptotic tumor cells induced by chemotherapeutic drugs to produce drug-packaging EVs [164]. Several anti-cancer drugs, including methotrexate, doxorubicin, and cisplatin, were shown to be packaged into EVs released by tumor cells, such as the mouse hepatocarcinoma tumor cell line H22 or the human ovarian cancer A2780. These drug-containing EVs effectively killed tumor cells in murine models without typical side effects, such as hair and/or weight loss or liver and/or kidney function impairment. Inspired by these preclinical results, three clinical trials were consecutively registered to test the effects of chemotherapeutic packed EVs in cancer patients (NCT01854866, NCT02657460, and NCT04131231). Whereas findings from two of the trials remain to be reported, one of the trials published their results in 2019 [163], showing that autologous tumor EVs packed with methotrexate symptomatically improved 10 of 11 lung cancer patients with malignant pleural effusion. The methotrexate-packed EVs activated antitumor effector cells including CTLs and TH1 in the pleural microenvironment and only caused mild (grades 1 to 2) adverse events.

Tumor EVs packed with chemotherapeutics also contributed to reverse drug resistance of malignant cells. Intrathoracic injection of cisplatin-packed tumor EVs in three end-stage lung cancer patients eliminated 95% of tumor cells in the malignant fluids and ameliorated patient symptoms. These therapeutic effects were absent in another three patients with intrathoracic injection of cisplatin alone [165].

### 5.4. Clinical Trials Using Plant-Derived sEVs

sEVs derived from plants are unquestionably safer than those from tumor cells. Grapefruits were found to yield higher sEVs (2.21 g/kg raw material) than grapes, tomatoes, bovine milk, or ginger [171]. Grapefruit-derived nanovectors (GNVs) were demonstrated to transport chemotherapeutic agents, siRNA, DNA expression vectors, and proteins to different kinds of cells. Co-delivery of folic acid and PTX by GNVs showed a therapeutic benefit in a mouse model of colon cancer [172]. These preclinical results led to a phase I clinical study investigating the efficacy of plant sEVs conjugated with curcumin that was orally delivered to patients with colon cancer (NCT01294072). Another phase I clinical trial was designed to evaluate the ability of plant sEVs to prevent oral mucositis during chemo-radiation of head and neck cancer (NCT01668849), which will shed light on the potential of using plant sEVs to alleviate side effects during cancer therapy.

### 5.5. Clinical Trials Using Normal Fibroblast-Like Mesenchymal Cell-Derived EVs

A preclinical study has demonstrated that sEVs, derived from fibroblast-like mesenchymal cells and loaded with siRNA or shRNA targeting KRAS mutation (KrasG12D), are significantly more effective than other drug carriers in inhibiting pancreatic ductal adenocarcinoma (PDAC) progression in vitro and in vivo [166]. Following the report, this research group initiated a phase I clinical trial (NCT03608631) aimed at testing this approach in patients with stage IV PDAC bearing the KrasG12D mutation. They will also evaluate median progression-free survival (PFS) and median overall survival (OS) as secondary objectives.

## 6. Conclusions

Research on EVs in cancer has been intensified over the last decade. The involvement of EVs, especially sEVs, in promoting cancer progression through intercellular communication is well recognized. This leads to efforts focusing on targeting EV signaling or utilizing EVs as drug carriers to develop novel cancer therapeutics. In this review, we have summarized recent progress in the development of EVs as cancer therapeutics, both in preclinical studies and clinical trials. Clearly, most of the studies reported on targeting sEV signaling, such as EV microRNA signaling, are at preclinical stages, and clinical trials are primarily related to developing EVs as therapeutic carriers at relatively early phases. This indicates that, on one hand, significant progress has been made in understanding how to better target EV signaling for the development of cancer therapeutics and the safety of delivering EVs into humans as therapeutic carriers, and on the other hand, clinical efficacy of EVs as therapeutic targets or therapeutic carriers remains to be determined. Compared to targeting EV signaling, utilizing EVs as therapeutic carriers seems to be a more practical strategy in therapeutic development and has advanced from preclinical studies to clinical trials. This is likely due to the fact that targeting cancer-specific EV signaling remains a challenge, as clear distinction of cancer EVs from healthy EVs has not been firmly established, and the heterogeneity of EVs is well recognized, which renders it difficult in specific targeting of EV signaling. In addition, current technology in EV isolation and validation needs to be improved, which also limits the effort in exploring EV signaling in cancer. Ongoing EV research needs to focus on these challenges in order to establish clinically applicable therapeutics targeting EV signaling in cancer. There are also challenges in the development of EVs as therapeutic carriers [173], including production and purification of EVs on an industrial scale, potential EV contamination with virus [100,174], and long-term side effects of tumor-derived EVs when they are applied as therapeutic carriers. However, these challenges are mostly technological, not conceptual, and hopefully can be overcome with concentrated effort. It is expected that EVs as therapeutic targets or delivery carriers may soon open up new avenues in clinical management of malignant diseases.

## Figures and Tables

**Table 1:** Clinical trials of EV-based cancer therapy.

Disease	Drug	EV Source	Phase, *n* of Patients	Status	Reference
Malignant Pleural Effusion	Methotrexate	Autologous Tumor-Derived Microparticles	Phase 2, *n =* 90	Recruiting	NCT02657460 ^1^ Guo, M. [163]
	Methotrexate	Microparticles	N/A, *n =* 248	Recruiting	NCT04131231 ^1^
	Chemotherapeutic Drugs	Tumor Cell- Derived Microparticles	Phase 2, *n =* 30	Unknown	NCT01854866 ^1^ Tang, K. [164]
	Cisplatin	Tumor Cell- Derived Microparticles	N/A, *n = 6*	Completed	Ma, J. [165]
Metastatic Pancreatic Cancer	KRAS ^2^ G12D siRNA	MSC ^3^-Derived Exosomes	Phase 1, *n =* 28	Recruiting	NCT03608631 ^1^ Kamerkar, S. [166]
Head and Neck Cancer	Grape Extract	Plant Exosomes	Phase 1, *n =* 60	Active, Not Recruiting	NCT01668849 ^1^
	Hemopurifier Pembro-lizumab	Blood-Derived Exosomes	N/A, *n =* 12	Not Yet Recruiting	NCT04453046 ^1^
Colorectal Cancer	Curcumin	Plant Exosomes	Phase 1, *n =* 7	Active, Not Recruiting	NCT01294072 ^1^
	GM-CSF ^4^	AEX ^5^	Phase 1, *n =* 40	Completed	Dai, S. [167]
Non-Small Cell Lung Cancer	Antigens	Tumor Dex2 ^6^	Phase 2, *n =* 41	Completed	NCT01159288 ^1^ Besse, B. [168]
	MAGE^7^ Tumor Antigens	Autologous DEX ^6^	Phase 1, *n =* 13	Completed	Morse, M.A. [169]
Metastatic Melanoma	MAGE^7^ 3 Peptides	Autologous DEX ^6^	Phase 1, *n* = 15	Completed	Escudier, B. [170]

^1^ The NCT# refers to a registered National Clinical Trial (NCT) which can be found at Clinicaltrials.gov, ^2^ Kirsten Rat Sarcoma (KRAS), ^3^ Mesenchymal Stem Cells (MSC), ^4^ Granulocyte- Macrophage Colony-Stimulating Factor (GM-CSF), ^5^ Ascites- Derived Exosomes (AEX), ^6^ Dendritic Cell- Derived Exosomes (DEX), ^7^ Melanoma Antigens (MAGE).

## Abbreviations

EV	Extracellular Vesicle
MV	Microvesicle
ISEV	International Society for Extracellular Vesicles
sEV	Small Extracellular Vesicle
MHC	Major Histocompatibility Complex
EPCAM	Epithelial Cell Adhesion Molecule
PECAM1	Platelet Endothelial Cell Adhesion Molecule 1
ERBB2	Erb-B2 Receptor Tyrosine Kinase 2
ESCRT	Endosomal Sorting Complexes Required for Transport
nSMase2	Neutral sphingomyelinase 2
HSP72	Heat-Shock Protein 72
STAT-3	Signal Transducer and Activator of Transcription 3
MA	Manumycin-A
PI3K	Phosphatidylinositol 3-Kinase
HUVEC	Human Umbilical Vein Endothelial Cell
GBM	Glioblastoma
CKAP4	Cytoskeleton-Associated Protein 4
DKK1	Dickkopf1
PDAC	Pancreatic Ductal Adenocarcinoma
PD1	Programmed Cell Death Protein 1
PD-L1	Programmed Death-Ligand 1
GMP	Good Manufacturing Practice
EPR	Enhanced Permeability and Retention
PSC	Pancreatic Stellate Cell
MSC	Mesenchymal Stem Cells
HeLa	Henrietta Lacks Cells
PTX	Paclitaxel
KRAS	Kirsten Rat Sarcoma
DEX	Dendrite Cell- Derived Exosomes
NSCLC	Non-Small Cell Lung Cancer
MAGE-3	Melanoma-Associated Antigen 3
IFN-γ-free DEX	Interferon Gamma-free Exosomes
IFN-γ-DEX	Interferon Gamma-containing Exosomes
AEX	Ascites-Derived Exosomes
GM-CSF	Granulocyte-Macrophage Colony-Stimulating Factor
CTL	Cytotoxic T Lymphocyte
TH1	T-cell Helper 1
GNV	Grapefruit-Derived Nanovectors
PFS	Progression-Free Survival
OS	Overall Survival
